# Adiposity, hormone replacement therapy use and breast cancer risk by age and hormone receptor status: a large prospective cohort study

**DOI:** 10.1186/bcr3186

**Published:** 2012-05-14

**Authors:** Rebecca Ritte, Annekatrin Lukanova, Franco Berrino, Laure Dossus, Anne Tjønneland, Anja Olsen, Thure Filskov Overvad, Kim Overvad, Françoise Clavel-Chapelon, Agnès Fournier, Guy Fagherazzi, Sabine Rohrmann, Birgit Teucher, Heiner Boeing, Krasimira Aleksandrova, Antonia Trichopoulou, Pagona Lagiou, Dimitrios Trichopoulos, Domenico Palli, Sabina Sieri, Salvatore Panico, Rosario Tumino, Paolo Vineis, José Ramón Quirós, Genevieve Buckland, Maria-José Sánchez, Pilar Amiano, María-Dolores Chirlaque, Eva Ardanaz, Malin Sund, Per Lenner, Bas Bueno-de-Mesquita, Carla H van Gils, Petra HM Peeters, Sanda Krum-Hansen, Inger Torhild Gram, Eiliv Lund, Kay-Tee Khaw, Nick Wareham, Naomi E Allen, Timothy J Key, Isabelle Romieu, Sabina Rinaldi, Afshan Siddiq, David Cox, Elio Riboli, Rudolf Kaaks

**Affiliations:** 1 Division of Cancer Epidemiology, German Cancer Research Center (DKFZ), Im Neuenheimer Feld TP4, Heidelberg, 69120, Germany; 2 Department of Preventive and Predictive Medicine, Fondazione IRCCS Istituto Nazionale dei Tumori, Via Venezian 1, Milan, 20133, Italy; 3 Institute of Cancer Epidemiology, Danish Cancer Society, Strandboulevarden 49, Copenhagen, 2100, Denmark; 4 Department of Cardiology, Aalborg Hospital, Aarhus University Hospital, Hobrovej 18-22, Aalborg, 9100, Denmark; 5 Department of Epidemiology, School of Public Health, Aarhus University, Bartholins Allé 2, Aarhus, 8000, Denmark; 6 Centre for Research in Epidemiology and Population Health, INSERM, Institut Gustave Roussy, 39 rue Camille Desmoulins, Villejuif, F-94805, France; 7 Paris South University, UMRS 1018, Villejuif, F-94805, France; 8 Division of Cancer Epidemiology and Prevention, Institute of Social and Preventive Medicine, Hirschengraben 84, Zurich, CH-8001, Switzerland; 9 Department of Epidemiology, German Institute of Human Nutrition, Potsdam-Rehbruecke, Arthur-Scheunert-Allee 114-116, Nuthetal, 14558, Germany; 10 WHO Collaborating Center for Food and Nutrition Policies, Department of Hygiene, Epidemiology and Medical Statistics, University of Athens Medical School, Asias Street 75 M., Goudi, Athens, GR-115 27, Greece; 11 Hellenic Health Foundation, Tetrapoleos Street 10-12, Athens, GR-115 27, Greece; 12 Department of Epidemiology, Harvard School of Public Health, Huntington Avenue 677, Boston, MA 02115, USA; 13 Bureau of Epidemiologic Research, Academy of Athens, Panepistimiou Street 28, Athens, GR-106 79, Greece; 14 Molecular and Nutritional Epidemiology Unit, Cancer Research and Prevention Institute (ISPO), Scientific Institute of Tuscany, Via Cosimo il Vecchio 2, Florence, 50139, Italy; 15 Nutritional Epidemiology Unit, Fondazione IRCCS Istituto Nazionale dei Tumori, Via Venezian1, Milano, 20133, Italy; 16 Department of Clinical and Experimental Medicine Medical School Federico II University Via S. Pansini 5 Naples, 80131, Italy; 17 Cancer Registry and Histopathology Unit, 'Civile M.P. Arezzo' Hospital ASP 7, Via Dante 109, Ragusa, 97100, Italy; 18 Centre for Environment and Health School of Public Health, Imperial College London, South Kensington Campus, London SW7 2AZ, UK; 19 Human Genetics Foundation (HuGeF), Via Nizza 52, Torino,10126, Italy; 20 Public Health and Health Planning Directorate, C/Ciriaco Miguel Virgil 9, Asturias, CP 33006, Spain; 21 Unit of Nutrition, Environment and Cancer, Cancer Epidemiology Research Programme, Catalan Institute of Oncology (ICO-IDIBELL), Avda Gran Via 199-203, Barcelona, 08907, Spain; 22 Andalusian School of Public Health, Cuesta del Observatorio 4, Granada, E-1801, Spain; 23 CIBER de Epidemiología y Salud Pública (CIBERESP), C/Melchor Fernández Almagro 3-5, Madrid, 28029, Spain; 24 Public Health Division of Gipuzkoa, Institute BIO Donostia, Health Department, Basque Region, Avda de Navarra 4, Gipuzkoa, 20013, Spain; 25 Department of Epidemiology, Murcia Regional Health Authority, Ronda de Levante 11, Murcia, 30008, Spain; 26 Navarra Public Health Institute, Leyre 15, Pamplona, 31003, Spain; 27 Department of Surgery and Perioperative Sciences, Umeå University Hospital, Building 10:1 Umeå, SE-901 85, Sweden; 28 Department of Oncology and Radiation Sciences, Oncology, Umeå University Hospital, Building 6M, Umeå, SE-901 87, Sweden; 29 National Institute for Public Health and the Environment, Antonie van Leeuwenhoeklaan 9, Bilthoven, 3721 MA, The Netherlands; 30 Department of Gastroenterology and Hepatology, University Medical Centre, Heidelberglaan 100, Utrecht, 3584 CX, The Netherlands; 31 Julius Center for Health Sciences and Primary Care, University Medical Center, Heidelberglaan 100, Utrecht, 3508 GA, The Netherlands; 32 Department of Epidemiology and Biostatistics, School of Public Health, Faculty of Medicine, Imperial College, London, South Kensington Campus, London SW7 2AZ, UK; 33 Institute of Community Medicine, University of Tromsø, MH Building, Tromsø, 9037, Norway; 34 School of Clinical Medicine, University of Cambridge, The Old Schools, Trinity Lane, CB2 1TN, UK; 35 Medical Research Council, Epidemiology Unit, Addenbrooke's Hospital, Hills Road, Cambridge, CB2 0QQ, UK; 36 Cancer Epidemiology Unit, University of Oxford, Richard Doll Building, Oxford, OX3 7LF, UK; 37 Nutritional Epidemiology Group, Section of Nutrition and Metabolism, International Agency for Research on Cancer (IARC), 150 Cours Albert Thomas, Lyon, 69372, France; 38 Department of Genomics of Common Disease, School of Public Health, Imperial College London, South Kensington Campus, London SW7 2AZ, UK

## Abstract

**Introduction:**

Associations of hormone-receptor positive breast cancer with excess adiposity are reasonably well characterized; however, uncertainty remains regarding the association of body mass index (BMI) with hormone-receptor negative malignancies, and possible interactions by hormone replacement therapy (HRT) use.

**Methods:**

Within the European EPIC cohort, Cox proportional hazards models were used to describe the relationship of BMI, waist and hip circumferences with risk of estrogen-receptor (ER) negative and progesterone-receptor (PR) negative (*n *= 1,021) and ER+PR+ (*n *= 3,586) breast tumors within five-year age bands. Among postmenopausal women, the joint effects of BMI and HRT use were analyzed.

**Results:**

For risk of ER-PR- tumors, there was no association of BMI across the age bands. However, when analyses were restricted to postmenopausal HRT never users, a positive risk association with BMI (third versus first tertile HR = 1.47 (1.01 to 2.15)) was observed. BMI was inversely associated with ER+PR+ tumors among women aged ≤49 years (per 5 kg/m^2 ^increase, HR = 0.79 (95%CI 0.68 to 0.91)), and positively associated with risk among women ≥65 years (HR = 1.25 (1.16 to 1.34)). Adjusting for BMI, waist and hip circumferences showed no further associations with risks of breast cancer subtypes. Current use of HRT was significantly associated with an increased risk of receptor-negative (HRT current use compared to HRT never use HR: 1.30 (1.05 to 1.62)) and positive tumors (HR: 1.74 (1.56 to 1.95)), although this risk increase was weaker for ER-PR- disease (*P*_*he*t _= 0.035). The association of HRT was significantly stronger in the leaner women (BMI ≤22.5 kg/m^2^) than for more overweight women (BMI ≥25.9 kg/m^2^) for, both, ER-PR- (HR: 1.74 (1.15 to 2.63)) and ER+PR+ (HR: 2.33 (1.84 to 2.92)) breast cancer and was not restricted to any particular HRT regime.

**Conclusions:**

An elevated BMI may be positively associated with risk of ER-PR- tumors among postmenopausal women who never used HRT. Furthermore, postmenopausal HRT users were at an increased risk of ER-PR- as well as ER+PR+ tumors, especially among leaner women. For hormone-receptor positive tumors, but not for hormone-receptor negative tumors, our study confirms an inverse association of risk with BMI among young women of premenopausal age. Our data provide evidence for a possible role of sex hormones in the etiology of hormone-receptor negative tumors.

## Introduction

Breast cancer is a complex and heterogeneous disease with a variety of histopathological and molecular subforms with diverse clinical outcomes and relationships with established risk factors [1,2]. One important subclassification of clinical breast tumors is based on the presence or absence of estrogen (ER) and progesterone (PR) receptors, and the routine identification of these receptors currently guides targeted therapies and provides important prognostic information [3]. The expression of the hormone receptors also broadly overlaps with more detailed molecular subclassifications of breast tumors as determined by microarray-based gene expression profiling [4,5].

Epidemiological data indicate that associations between excess body mass index (BMI) and the risk of breast cancer may differ by the ER and PR status in breast tumors [6], and that the positive association of excess adiposity with breast cancer risk after menopause may be driven predominantly by the association with receptor-positive (ER+ or ER+PR+) disease [6]. For receptor-negative breast tumors, the association of risk with excess weight is less well characterized, and studies have shown variable and somewhat conflicting results [6-9]. In part, the diversity of previous findings could be related to heterogeneity in the association of excess weight with breast cancers diagnosed at a predominantly premenopausal or more advanced postmenopausal ages.

Another important factor to be accounted for when assessing the relationships of excess adiposity with postmenopausal breast cancer risk is use of hormone replacement therapy (HRT). Various studies have shown interactions of BMI with postmenopausal HRT use as risk factors for breast cancer [6,10-14]. However these interactions have not been described for hormone receptor-defined breast cancer subtypes.

The present analysis extends on a previous report (2004) from the European Prospective Investigation into Cancer and Nutrition (EPIC) in which the relationships of anthropometry with risk of total breast cancer were investigated [14]. Using more recent follow-up data from EPIC, which now includes a total of 7,174 incident cases of breast cancer with information on ER status and 5,906 with additional PR status, we examined the relationships of anthropometric indices of adiposity (BMI, waist and hip circumferences) with risks of breast cancer subtypes defined by hormone receptor status, across five-year age bands spanning from premenopausal into postmenopausal years, and accounting for past or current use of menopausal HRT.

## Materials and methods

EPIC is a multi-center prospective cohort study designed to investigate the relationships between diet, nutrition and metabolic factors and cancer, consisting of about 370,000 women and 150,000 men aged mostly between 25 and 70 years [15,16]. All participants were enrolled between the years 1992 and 2000 from 23 regional and national research centers located in 10 western European countries: Denmark, France, Germany, Greece, Italy, The Netherlands, Norway, Spain, and the United Kingdom.

Extensive details about the standardized procedures for recruitment, measuring baseline anthropometry (height, weight, waist and hip circumferences), questionnaires on current habitual diet, reproductive and menstrual history, exogenous hormone use (oral contraceptive (OC) and HRT use), medical history, lifetime smoking and alcohol consumption history, occupation, level of education and physical activity and biological sample collection at study centers are given elsewhere [15,16]. All subjects gave written informed consent to use their questionnaire data and the Internal Review Boards (IRB) of International Agency for Research on Cancer (IARC) and all EPIC recruitment centers approved the analyses based on EPIC participants.

### Study participants

Of the 367,903 female participants in EPIC, women were *a priori *excluded if they had a history of cancer prior to recruitment (*n *= 19,853) or were missing a diagnosis or censoring date (*n *= 2,892), thus leaving 345,158 participants. Three EPIC study centers did not provide any information on breast tumor receptor status and, therefore, were also systematically excluded from this analysis (*n *= 26,091). Women were further excluded if they were missing questionnaire data (*n *= 526) or data on height and weight (*n *= 3,865). This left a total cohort of 314,676 women from 10 countries for our present analysis.

### Classification of body measurements and baseline variables

The details of standardized procedures for measuring anthropometry and baseline variables at study centers have previously been reported [17]. Height, waist and hip circumferences were measured to the nearest centimeter and weight to the nearest kilogram. Most subjects had their height, weight, hip and waist circumferences measured in light clothing and without shoes. Waist circumference was measured by either the narrowest torso circumference or midway between the lower ribs and the iliac crest. The hip circumference was measured from either the widest point or over the buttocks. Body mass index (BMI) was constructed by dividing weight by height in meters squared (kg/m^2^). In Norway, Umea and a large proportion of the EPIC cohort in France (69%) subjects' height and weight were measured and self-reported by the cohort participants themselves, following detailed instructions [16,17]. Waist and hip circumferences measurements were not collected from these centers. In Oxford, subjects also self-reported their height, weight, and waist and hip circumferences; however, a subset of these participants were also measured by study researchers in order to calibrate self-reported measurements of the Oxford cohort [18].

Women were considered postmenopausal at recruitment if they had had no menstrual cycles in the last 12 months, were older than 55 years (if the menstrual cycle history was missing), or had a bilateral oophorectomy. Women who reported having had a hysterectomy but still had either one or both ovaries intact had their menopausal status classified using their reported menstrual history, age and exogenous hormone use. Women were classified with a peri/-or of unknown menopausal status if they had experienced one to nine menstrual cycles in the last 12 months, were aged 46 to 55 years if menstrual cycle history was missing, or were taking HRT at the time of recruitment. Women were deemed premenopausal if they had had ≥10 menstrual cycles in the last 12 months or were younger than 46 years if the menstrual cycle history was missing. Women who were < 46 years of age, still had one or both ovaries and were still menstruating were considered as premenopausal if they indicated exogenous hormone use.

Information on postmenopausal hormone use was derived from country-specific baseline questionnaires covering ever and current use of HRT, brand name used at recruitment and age at start and duration of use [19]. Of the women who reported HRT ever or current use at baseline, subgroups of different HRT regimes were created (current estrogen only or current estrogen plus progestin users (estrogen with sequential or continuous daily use of progestin)). Duration of HRT use and time since last use (created by combining information of duration of past use and age at starting HRT) was restricted to information collected at baseline recruitment.

### Perspective ascertainment of breast cancer cases and the coding of receptor status

In all countries (except for France, Germany and Greece) incident breast cancer cases were identified using record linkage with cancer and pathology registries. In France, Germany, and Greece, cancer occurrence was prospectively ascertained through linkage with health insurance records and regular direct contacts with participants and their next of kin, and all reported breast cancer cases were then systematically verified against clinical and pathological records. Cancer incidence data were classified according to the International Classification of Diseases, 10^th ^Revision (ICD-10). Information on tumor receptor status, as well as the available laboratory methods and quantification descriptions used to determine receptor status was collected by 20 centers. To standardize the quantification of receptor status received from the EPIC centers, the following criteria for a positive receptor status were used; ≥10% cells stained, any 'plus-system' description, ≥20 fmol/mg, an Allred score of ≥3, an IRS ≥2, or an H-score ≥10 [20-24].

Vital status was collected from regional or national mortality registries. The last updates of endpoint data for cancer incidence and vital status were between 2005 and 2010, depending on the center. Women were considered at risk from the time of recruitment until breast cancer diagnosis or censoring (age at death, loss to follow-up, end of follow-up, or diagnosis of other cancer cases). Three EPIC subjects had the same date of recruitment and date of exit and were excluded because they did not contribute to the underlying time at risk variable. An additional three subjects with a breast cancer diagnosis were excluded because it was unclear whether their diagnosis was a primary incidence case, thus a total of 9,530 breast cancer cases were included for this analysis. A total of 7,174 breast cancer cases had information on ER status (5,764 ER-positive, 1,410 ER-negative); of which, 5,906 had further information on PR status (3,586 ER+PR+, 1,086 ER+PR-, 213 ER-PR+, 1,021 ER-PR-).

### Statistical analysis

Correlations between indices of adiposity were assessed using Pearson's partial correlation adjusted for study center and age at recruitment. Associations between BMI, waist and hip circumferences and the risk of breast cancer subtype were evaluated using Cox proportional hazards models to estimate hazard ratios (HR) and 95% confidence intervals (CI), with total breast cancer cases and breast cancer subtype outcomes defined as either jointly classified ER+PR+, ER+PR-, ER-PR+, ER-PR- or missing receptor status, ER-positive, ER-negative or missing receptor status, or, PR-positive, PR-negative or missing receptor status. All multivariable analyses were stratified by age in one-year categories and by study center, to prevent violations of the proportional-hazards assumption.

To analyze associations of BMI, waist and hip circumferences with risks of breast cancer subtypes in all women, age bands were created by counting person years within each age limit, with left and/or right side censoring age limits defined as 'less than or equal to 49 years', 'between 50 and 54 years', 'between 55 and 59 years', 'between 60 and 64 years' and '65 years and older'. Incidence rates of breast cancer subtypes were calculated from person years and observed numbers of incident cases within age bands. Anthropometric measures were examined across the age bands on a continuous scale.

BMI has been observed to interact with postmenopausal HRT use [11,14,25], and to eliminate any possible interaction effects, the association of BMI, waist and hip circumferences were analyzed within the group of HRT never users only. Differences in recording baseline HRT use across the EPIC centers caused difficulties in establishing the reliability of baseline HRT use for younger women (less than 55 years at recruitment) who were still pre- or perimenopausal. Therefore, we restricted the analyses by age bands for baseline never users of HRT to women aged 55 years and over. Furthermore, possible interactions between HRT use (never, past and current) and BMI were assessed with respect to risks of the breast cancer subtypes among postmenopausal women only.

In postmenopausal women, BMI tertiles were created using cut-points based on the overall cohort distribution of body measurements (BMI tertile 1: ≤22.5 kg/m^2^; BMI tertile 2: 22.6 to 25.8 kg/m^2^; BMI tertile 3: ≥25.9 kg/m^2^). Combined risk categories of BMI (tertiles) and HRT use were constructed using never users with a BMI in the lowest tertile as the reference category. Statistical interaction between categories of HRT use (never versus current use) and the integer score of BMI tertiles (1,2,3) entered as an ordered, quantitative variable was assessed by including an interaction term and the log likelihood ratio test.

A multivariable model stratified by center and age at recruitment with further adjustments for age at menarche (less than 11 years, 11 to 14 years and greater than 14 years, missing), age at first child birth (less than 20 years, 20 to 30 years, greater than 30 years, missing), parity (nulliparous, one full-term birth, two full-term births or three or more full-term births, missing), history of breastfeeding (ever versus never, missing), use of oral contraceptives (OC) (ever versus never, missing), smoking status (current, former, never, missing), alcohol consumption (nonconsumers (< 1.5 g/day), 1.5 g to 10 g/day (as the reference category), 10 to 20 g/day, 20 to 30 g/day and greater than 30 g/day, missing), physical activity (Cambridge Index: active, moderately active, moderately inactive and inactive, missing [26]), education level (none, primary school, technical/professional school, secondary school, university degree or equivalent, missing), HRT use (ever versus never, missing) and age at menopause in postmenopausal women only was assessed. Both the reduced model stratified by age and center and the multivariable model are presented; however, as the risk estimates were very similar only the reduced model results are discussed. In addition, adjustments for BMI (as a continuous variable) were used to examine whether measures of body distribution (waist circumference, hip circumference) were associated with breast cancer risk independently of general excess weight.

Differences between BMI, waist and hip circumferences and risk of breast cancer subtypes were analyzed using the data augmentation method as described by Lunn and McNeil, using a log likelihood ratio test to compare the model with and without interaction terms between the anthropometric variable and breast cancer subtype [27]. Women who developed the competing breast cancer subtype or were missing receptor status were also censored at the time of occurrence [27].

A sensitivity analysis that excluded all uncalibrated self-reported anthropometric measurements to assess the effect of including self-reported indices of BMI, waist and hip circumferences into the models found no difference between how measurements of anthropometry were obtained (data not shown) so, therefore, all self-reported anthropometric measurements were retained in the models. All tests of significance were two-sided and all analyses were completed using SAS version 9.2; SAS Institute, Cary, NC.

## Results

A total cohort of 314,760 women was followed for a sum of 3,399,178 person years. Of these, 144,223 (45.8%) were postmenopausal at the time of recruitment, of which 93.6% had a natural menopause. Baseline characteristics of all women and postmenopausal women within this cohort are presented in Table 1. The median age at recruitment from the EPIC centers was 50.9 years for all women, 58.1 for women who were postmenopausal, 42.6 for women who were premenopausal and 50.1 for women whose menopausal status was either perimenopausal or undetermined at baseline. Of the postmenopausal women who provided information on HRT use, 42,719 (29.6%) were current users at recruitment, 18,278 (12.7%) were past users and 78,919 (54.7%) were never users. Postmenopausal women of higher degrees of BMI were more likely to be never users of HRT and had a shorter duration of past HRT use (Additional file 1 Table S1).

**Table 1:** EPIC cohort characteristics for all women and postmenopausal women at baseline recruitment, 2010.

	All women	Postmenopausal women	BMI tertiles^6^		
			1	2	3
	(*n *= 314760)	(*n *= 144223)	(*n *= 36792)	(*n *= 48846)	(*n *= 58585)
**Person years**	3399178	1542924	391812	525727	625386
**Age at recruitment^1^**	50.9(19.9-98.5)	58.1(27.8-98.5)	56.8(32.4-98.5)	57.8(32.3-94.9)	59.1(27.8-88.6)
**Age at exit^1^**	61.9(21.1-102.4)	68.9(40.4-102.4)	67.7(40.4-102.4)	68.7(40.8-101.3)	69.8(40.5-98.3)
**Years of follow-up^2^**	11.4(0.01-16.8)	11.5(0.01-16.7)	11.6(0.01-16.7)	11.6(0.02-16.6)	11.5(0.01-16.6)
**Years until diagnosis^3^**	6.1(0.01-15.6)	6.0(0.01-15.3)	5.8(0.01-14.4)	6.0(0.02-14.7)	6.0(0.01-15.3)
**BMI (kg/m^2^)^1^**	24.02(10.2-77.9)	24.8(12.9-77.9)	21.1(12.9-22.5)	24.1(22.6-25-8)	28.8(25.9-77.9)
**Hip circumference (cm)**	100.0(50.0-179.0)	81.0(51.0-179.0)	71.0(52.0-117.0)	78.0(51.0-171.0)	90.0(52.0-179.0)
**Waist circumference (cm)**	78.0(50.0-179.0)	101.0(53.0-171.0)	93.0(57.0-115.0)	99.0(55.0-158.0)	107.5(53.0-171.0)
**Premenopausal^4^**	35.5%(111560)	-	-	-	-
**Peri/or of unknown menopausal status^4^**	18.7%(58887)	-	-	-	-
**Postmenopausal^4^**	45.8%(144223)	-	-	-	-
**Never HRT user^5^**	38.4%(120992)	54.7%(78,919)	45.0%(16569)	51.4%(25108)	63.6%(37242)
**Past HRT user^5^**	7.0%(21980)	12.7%(18,278)	12.6%(4640)	13.1%(6401)	12.4%(7237)
**Current HRT user^5^**	16.5%(51975)	29.6%(42,719)	39.8%(1462)	32.4%(15826)	20.9%(12266)
**Missing HRT use^5 ^**	38.0%(119723)	3%(4,307)	2.6%(956)	3.1%(1511)	3.1%(1840)

^1^Median (range); ^2^reverse Kaplan Meier median (range); ^3^in primary incident cases only; ^4^percentage (number); ^5^excluding premenopausal women at baseline recruitment; ^6^in postmenopausal women at baseline recruitment only. BMI, body mass index; HRT, hormone replacement therapy.

For the great majority of postmenopausal women who reported HRT current use and had information of HRT regimen (*n *= 35,856 (76.0%)), combinations of estrogen plus progestin were used, while almost one out of four HRT users (24.0%) used formulae based on estrogens alone. Almost half of all breast cancer cases (46.2%) were diagnosed at the age of 60 years or older. The median time until diagnosis was 6.1 years for all incident cases, 6.3 years for ER-PR- tumors, 6.8 years for ER+PR+ tumors, and 5.0 years for tumors with unknown ER or PR receptor status. Incident cases with ER-PR- breast tumors were rather evenly distributed across the age bands, whereas the majority of cases with ER+PR+ breast tumors were diagnosed after the age of 55 (Table 2). A basic calculation of incidence rates across the five-year age bands showed that ER-PR- breast cancer rates were stable across all age bands above age 50 (for ≤49 years: 16.9, 50 to 54 years: 34.6, 55 to 59 years: 36.9, 60 to 64 years: 38.4, ≥65 years: 29.1 per 100,000 person years), whereas ER+PR+ disease, rates increased steadily with increasing age albeit at a slower rate after 50 years (for ≤49 years: 44.5, 50 to 54 years: 111.2, 55 to 59 years: 125.4, 60 to 64 years: 142.6, ≥65 years: 127.9 per 100,000 person years). [28]

**Table 2:** Cohort size, person years and incident cases of breast cancer subtypes across five-year age bands.

	Age bands										
	≤ 49 years		50-54 years		55-59 years		60-64 years		≥ 65 years		All women
**Subjects at risk**	141513		175268		185404		161850		113810		314670
**Person years**	870491		627875		661652		565289		673872		3399178
											
**Total cases^1^**	1205	(12.6%)	1770	(18.6%)	2151	(22.6%)	2065	(21.7%)	2339	(24.5%)	9530
											
**ER-positive**	507	(8.8%)	1031	(17.9%)	1402	(24.3%)	1345	(23.3%)	1479	(25.7%)	5764
**ER-negative**	235	(16.7%)	306	(21.7%)	320	(22.7%)	294	(20.9%)	255	(18.1%)	1410
**ER-missing**	463	(19.7%)	433	(18.4%)	429	(18.2%)	426	(18.1%)	605	(25.7%)	2356
											
**PR-positive**	462	(12.1%)	755	(19.8%)	872	(22.9%)	839	(22.0%)	880	(23.1%)	3808
**PR-negative**	212	(10.0%)	397	(18.8%)	553	(26.2%)	499	(23.6%)	453	(21.4%)	2114
**PR-missing**	531	(14.7%)	618	(17.1%)	726	(20.1%)	727	(20.1%)	1,006	(27.9%)	3608
											
**ER+PR+**	390	(10.9%)	698	(19.5%)	830	(23.1%)	806	(22.5%)	862	(24.0%)	3586
**ER+PR-**	64	(5.9%)	180	(16.6%)	306	(28.2%)	280	(25.8%)	256	(23.6%)	1086
**ER-PR+**	71	(33.3%)	57	(26.8%)	37	(17.4%)	31	(14.6%)	17	(8.0%)	213
**ER-PR-**	147	(14.4%)	217	(21.3%)	244	(23.9%)	217	(21.3%)	196	(19.2%)	1021
**ER or PR missing**	533	(14.7%)	618	(17.1%)	734	20.3%	731	(20.2%)	1008	(27.8%)	3624

^1^Including women of unknown receptor status. ER, estrogen receptor; PR, progesterone receptor.

**Figure 1:**
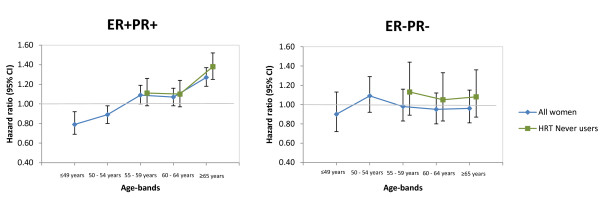
**Hazard ratios of ER+PR+ and ER-PR- tumors for increases in BMI across age bands**. All models are for a 5 kg/m^2 ^increase in BMI and were stratified by age at recruitment and study center. Hazard ratio estimates are shown for all women and HRT never users. BMI, body mass index; ER, estrogen receptor; HRT, hormone replacement therapy; PR, progesterone receptor.

Across the age bands, Cox regression models showed no distinct association for BMI with risk of ER-PR- breast cancer (Figure 1 and Table 3). It was only among women older than 65 years that the relative risk for ER-PR- tumors (per five-unit increase in BMI HR = 0.95 (95%CI: 0.79 to 1.13)) was statistically different from ER+PR+ tumors (per five-unit increase in BMI HR = 1.25 (95%CI: 1.16 to 1.34); *P *< 0.001; *P_het _*= 0.004). For ER+PR+ tumors, the negative risk association for BMI among women aged less than 49 years (per five-unit increase in BMI HR = 0.79 (95%CI: 0.68 to 0.91); *P *= 0.002) progressively turned into a significant positive risk association among women aged 65 years and older.

**Table 3:** Hazard ratios of joint ER and PR tumors per 5 cm increase in BMI across each age band among all women and never users of HRT.

	≤49 years			Between 50 and 54 years			Between 55 and 59 years			Between 60 and 64 years			≥65 years		
	Cases	HR	95%CI	Cases	HR	95%CI	Cases	HR	95%CI	Cases	HR	95%CI	Cases	HR	95%CI
**All women**															
*Age and center stratified*															
ER+PR+	390	0.79	(0.68-0.91)	698	0.88	(0.79-0.97)	830	1.08	(0.69-1.06)	806	1.05	(0.96-1.14)	862	1.25	(1.16-1.34)
ER+PR-	64	1.10	(0.81-1.50)	180	0.86	(0.69-1.06)	306	0.86	(0.68-1.38)	280	0.86	(0.74-1.01)	256	0.92	(0.78-1.08)
ER-PR+	71	0.68	(0.47-0.99)	57	0.97	(0.68-1.38)	37	0.55	(0.91-1.27)	31	0.83	(0.50-1.37)	17	0.70	(0.35-1.42)
ER-PR-	147	0.89	(0.72-1.12)	217	1.08	(0.91-1.27)	244	0.98	(0.93-1.13)	217	0.95	(0.80-1.12)	196	0.95	(0.79-1.13)
ER or PR missing	533	0.92	(0.82-1.03)	618	1.03	(0.93-1.13)	734	1.03	(1.00-1.18)	731	1.02	(0.94-1.11)	1008	1.09	(1.01-1.17)
*P*_heterogeneity_^2^			0.36			0.05			0.26			0.28			0.004
															
*Multivariable model^4^*															
ER+PR+	390	0.80	(0.69-0.93)	698	0.90	(0.81-1.01)	830	1.11	(1.01-1.21)	806	1.10	(1.01-1.20)	862	1.32	(1.22-1.43)
ER+PR-	64	1.14	(0.83-1.55)	180	0.90	(0.72-1.12)	306	0.90	(0.76-1.05)	280	0.88	(0.75-1.03)	256	0.97	(0.82-1.14)
ER-PR+	71	0.67	(0.46-0.99)	57	0.98	(0.68-1.40)	37	0.57	(0.32-1.01)	31	0.84	(0.50-1.43)	17	0.79	(0.37-1.68)
ER-PR-	147	0.90	(0.71-1.13)	217	1.09	(0.92-1.29)	244	1.00	(0.84-1.18)	217	0.97	(0.81-1.16)	196	0.97	(0.81-1.17)
ER or PR missing	533	0.93	(0.83-1.05)	618	1.08	(0.98-1.19)	734	1.08	(0.99-1.18)	731	1.06	(0.97-1.16)	1008	1.13	(1.05-1.22)
*P*_heterogeneity_^2^			0.44			0.08			0.29			0.20			0.002
															
**HRT never users**															
*Age and center stratified*															
ER+PR+							350	1.10	(0.97-1.25)	348	1.08	(0.96-1.22)	441	1.34	(1.22-1.47)
ER+PR-							135	0.87	(0.69-1.10)	118	0.95	(0.76-1.19)	138	0.96	(0.78-1.18)
ER-PR+							16	0.63	(0.28-1.40)	10	0.74	(0.30-1.79)	8	0.98	(0.42-2.29)
ER-PR-							95	1.12	(0.88-1.42)	90	1.05	(0.83-1.33)	105	1.06	(0.85-1.32)
ER or PR missing							304	1.06	(0.94-1.21)	325	1.08	(0.96-1.22)	566	1.13	(1.03-1.24)
*P*_heterogeneity_^2^									0.94			0.83			0.05
															
*Multivariable model^5^*															
ER+PR+							350	1.11	(0.97-1.27)	348	1.13	(1.00-1.28)	441	1.38	(1.25-1.52)
ER+PR-							135	0.91	(0.72-1.15)	118	0.96	(0.76-1.21)	138	1.02	(0.83-1.25)
ER-PR+							16	0.62	(0.26-1.49)	10	0.63	(0.24-1.67)	8	1.06	(0.37-3.03)
ER-PR-							95	1.19	(0.93-1.54)	90	1.09	(0.85-1.40)	105	1.11	(0.88-1.39)
ER or PR missing							304	1.09	(0.96-1.24)	325	1.11	(0.98-1.25)	566	1.17	(1.06-1.28)
*P*_heterogeneity_^2^									0.62			0.83			0.08

^1^Per 5 kg/m^2 ^increase; ^2^heterogeneity between ER+PR+ versus ER-PR- tumors using data augmentation method as described by Lunn and McNeil; ^3^multivariable hazard ratios were stratified for study center, age, and adjusted for height (cm), educational attainment, smoking status, alcohol consumption, parity, age at first pregnancy, age at menarche, menopausal status, age at menopause (in postmenopausal women), ever OC use and current HRT use; ^4^multivariable hazard ratios were stratified for study center, age, and adjusted for height (cm), educational attainment, smoking status, alcohol consumption, parity, age at first pregnancy, age at menarche, ever OC use, menopausal status, age at menopause (in postmenopausal women). BMI, body mass index; CI, confidence interval; ER, estrogen receptor; HR, hazard ratio; HRT, hormone replacement therapy; PR, progesterone receptor.

Restricting the analysis to women who never used HRT, a five-unit increase in BMI showed a weak tendency towards a risk of ER-PR- disease; although this trend was not statistically significant across any age band. There was an indication of heterogeneity between the risks estimates of ER-PR- (per five-unit increase in BMI HR = 1.06 (0.85 to 1.32)) and ER+PR+ tumors (per five-unit increase in BMI HR = 1.34 (1.22 to 1.47)) among the HRT never users older than 65 years (*P*_het _= 0.05). Similar patterns of association were observed for BMI with risk of ER-negative versus ER-positive breast cancer subtypes, ignoring PR status (Additional file 2, Figure S1), and as well as PR-negative versus PR-positive breast cancer subtypes, ignoring ER status (Additional file 3, Figure S2).

Increased waist or hip circumferences showed relative risk patterns across the age bands that were very similar to those for BMI, for both ER+PR+ and ER-PR- breast cancer subtypes, among all women as well as among current and past users of HRT (Additional files 4 and 5, Figures S3 and S4). After adjustment for BMI, however, neither waist nor hip circumferences showed any relationship with risks of either breast cancer subtype (data not shown). Pearson's correlations showed that increases in BMI were highly correlated with increases in both waist and hip circumferences (Pearson's correlations coefficient r = 0.83 and 0.85 respectively), therefore this could account for the lack of associations observed of waist and hip circumferences after adjustment for BMI.

With regard to postmenopausal HRT use, Cox regression models showed that in comparison to HRT never users, postmenopausal women who were current HRT users at baseline had significantly increased risks of both ER-PR- breast cancer (HR = 1.30 (95%CI: 1.05 to 1.62); *P*_value _= 0.02) and ER+PR+ breast cancer (HR = 1.74 (95%CI: 1.56 to 1.95); *P*_value _< 0.001), although the relative risk was significantly weaker for ER-PR- than for ER+PR+ tumors (*P*_het _= 0.035).

**Figure 2:**
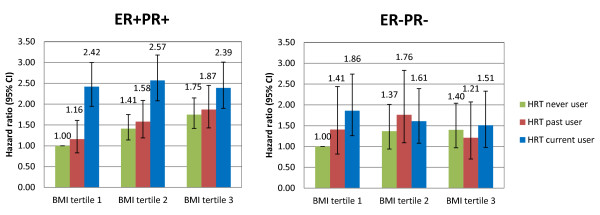
**Hazard ratios of ER+PR+ and ER-PR- tumors for increases in BMI across HRT user categories**. All models were restricted to postmenopausal women with information on baseline HRT use and stratified by age at recruitment and study center. HRT never users within BMI tertile1 were used as the reference category. BMI tertile 1: ≤22.5 kg/m^2^; BMI tertile 2: 22.6 to 25.8 kg/m^2^; BMI tertile 3: ≥25.9 kg/m^2^. BMI, body mass index; ER, estrogen receptor; HRT, hormone replacement therapy; PR, progesterone receptor.

For combined risk categories determined by the joint classification of HRT use (never, past, current) and BMI tertiles, for ER-PR- breast cancer, using never users of HRT in the lowest BMI tertile as a reference category, the highest relative risk for current HRT use was seen for women in the first BMI tertile, while relative risks were somewhat weaker for women in the middle and upper tertiles of BMI (Figure 2). Within separate categories of HRT use, we observed a positive risk association within the third tertile of BMI with ER-PR- breast cancer among never users of HRT (third versus first tertile HR = 1.47 (95%CI: 1.01 to 2.15); *P*_trend _= 0.06) (Table 4) contrasted by an indication of a negative risk association among current and no association with past users of HRT. Switching perspective and stratifying the analysis by separate strata of BMI, the relative risk for current HRT use was strongest for women within the first BMI tertile and absent for women in the higher tertiles. This interaction of the effects of HRT use (current versus never users) across BMI tertile scores was statistically significant (*P*_interaction _= 0.037) (Table 5) and was not restricted to HRT type (Additional files 6, 7, 8, 9, 10 and 11, Tables S2 to S5 and Figures S5 and S6).

**Table 4:** Hazard ratios of ER+PR+ and ER-PR- tumors across levels of BMI within categories of postmenopausal HRT users.

HRT use	BMI tertile^1^										
	1		2			3				*Per 5 kg/m^2 ^increase*	
	*Cases*	Reference	*Cases*	HR	95% CI	*Cases*	HR	95% CI	*Cases*		
***ER+PR+***											
***Age and center stratified***											
Never user	*125*	1.00	*248*	1.44	(1.15-1.78)	*392*	1.80	(1.46-2.23)	*765*	1.24	(1.15-1.34)
Past user	*49*	1.00	*81*	1.45	(1.01-2.09)	*95*	1.85	(1.27-2.69)	*225*	1.45	(1.25-1.68)
Current user	*296*	1.00	*312*	1.03	(0.87-1.21)	*200*	0.74	(0.50-1.09)	*808*	0.99	(0.89-1.09)
											
***ER+PR+***											
***Multivariable model^2^***											
Never user	*125*	1.00	*248*	1.47	(1.18-1.82)	*392*	1.90	(1.53-2.35)	*765*	1.28	(1.18-1.38)
Past user	*49*	1.00	*81*	1.43	(0.99-2.07)	*95*	1.89	(1.29-2.77)	*225*	1.47	(1.26-1.72)
Current user	*296*	1.00	*312*	1.04	(0.88-1.23)	*200*	0.95	(0.78-1.15)	*808*	1.01	(0.91-1.12)
											
***ER-PR-***											
***Age and center stratified***											
Never user	*41*	1.00	*80*	1.40	(0.96-2.06)	*103*	1.47	(1.01-2.15)	*224*	1.07	(0.93-1.25)
Past user	*19*	1.00	*29*	1.20	(0.66-2.20)	*69*	0.80	(0.40-1.60)	*117*	0.86	(0.62-1.19)
Current user	*79*	1.00	*69*	0.82	(0.59-1.15)	*45*	0.74	(0.50-1.09)	*193*	0.79	(0.63-0.98)
											
***ER-PR-***											
***Multivariable model***											
Never user	*41*	1.00	*80*	1.44	(0.98-2.11)	*103*	1.59	(1.08-2.34)	*224*	1.12	(0.96-1.31)
Past user	*19*	1.00	*29*	1.18	(0.64-2.18)	*69*	0.76	(0.37-1.56)	*117*	0.82	(0.58-1.16)
Current user	*79*	1.00	*69*	0.82	(0.59-1.15)	*45*	0.77	(0.52-1.14)	*193*	0.80	(0.64-1.01)

^1^BMI tertile 1: ≤22.5 kg/m^2^; BMI tertile 2: 22.6 to 25.8 kg/m^2^; BMI tertile 3: ≥25.9 kg/m^2^; ^2 ^multivariable hazard ratios were stratified for study center, age, and adjusted for height (cm), educational attainment, smoking status, alcohol consumption, parity, age at first pregnancy, age at menarche, ever OC use, menopausal status, age at menopause. BMI, body mass index; CI, confidence interval; ER, estrogen receptor; HR, hazard ratio; HRT, hormone replacement therapy; PR, progesterone receptor.

**Table 5:** Hazard ratios of ER+PR+ and ER-PR- tumors for categories of postmenopausal HRT use within tertiles of BMI.

**BMI tertile**^3^	HRT use								
	Never user		Past user			Current user			
	*Cases*	Reference	*Cases*	HR	95% CI	*Cases*	HR	95% CI	*P_interaction_^2^*
***ER+PR+***									
***Age and center stratified***									
BMI tertile 1	*125*	1.00	*49*	1.18	(0.84-1.65)	*296*	2.33	(1.84-2.92)	*< 0.001*
BMI tertile 2	*248*	1.00	*81*	1.10	(0.85-1.42)	*312*	1.75	(1.45-2.12)	
BMI tertile 3	*392*	1.00	*95*	1.11	(0.88-1.39)	*200*	1.45	(1.19-1.77)	
									
***ER+PR+***									
***Multivariable model^3^***									
BMI tertile 1	*125*	1.00	*49*	1.17	(0.83-1.64)	*296*	2.32	(1.84-2.92)	*< 0.001*
BMI tertile 2	*248*	1.00	*81*	1.10	(0.85-1.42)	*312*	1.75	(1.45-2.11)	
BMI tertile 3	*392*	1.00	*95*	1.11	(0.88-1.39)	*200*	1.45	(1.19-1.76)	
									
***ER-PR-***									
***Age and center stratified***									
BMI tertile 1	*41*	1.00	*19*	1.38	(0.79-2.43)	*79*	1.74	(1.15-2.63)	*0.037*
BMI tertile 2	*80*	1.00	*29*	1.20	(0.78-1.86)	*69*	1.07	(0.75-1.54)	
BMI tertile 3	*103*	1.00	*20*	0.92	(0.57-1.51)	*45*	1.21	(0.81-1.81)	
									
***ER-PR-***									
***Multivariable model***									
BMI tertile 1	*41*	1.00	*19*	1.38	(0.79-2.43)	*79*	1.74	(1.15-2.63)	*0.037*
BMI tertile 2	*80*	1.00	*29*	1.18	(0.77-1.83)	*69*	1.08	(0.75-1.55)	
BMI tertile 3	*103*	1.00	*20*	0.92	(0.57-1.51)	*45*	1.21	(0.81-1.81)	

^1^BMI tertile 1: ≤22.5 kg/m^2^; BMI tertile 2: 22.6 to 25.8 kg/m^2^; BMI tertile 3: ≥25.9 kg/m^2^; ^2^*P *for interaction was calculated using the log likelihood ratio test for models with and without the interaction term for HRT never and current use by BMI tertiles; ^3^multivariable hazard ratios were stratified for study center, age, and adjusted for height (cm), educational attainment, smoking status, alcohol consumption, parity, age at first pregnancy, ever OC use, age at menarche, menopausal status, age at menopause. BMI, body mass index; CI, confidence interval; ER, estrogen receptor; HR, hazard ratio; HRT, hormone replacement therapy; PR, progesterone receptor.

For ER+PR+ breast cancer, compared to never users of HRT in the lowest BMI tertile as a reference category, we observed an approximate 2.5-fold increase in risk for ER+PR+ breast cancer for baseline HRT users in each of the three BMI tertiles when we used never users in the lowest BMI tertile as the reference category (Figure 2). However, performing analyses within independent strata for baseline HRT use (never, past, current), our data showed more than a 1.8-fold increase in risk for the highest BMI tertile compared to the lowest tertile in the strata of never and past users of HRT, but no such association at all among current HRT users (Table 4). Alternatively, examining relative risks for HRT use within strata of each of the BMI tertiles separately, the relative risk for ER+PR+ breast cancer associated with current use of HRT was strongest for women within the lowest BMI tertile, and progressively weakened across BMI tertiles 2 and 3, respectively (*P*_interaction _< 0.001)(Table 5).

## Discussion

Overall, for ER-PR- tumors, our study showed no distinct associations with BMI in any of the five-year age bands. However, among postmenopausal women who never used HRT, our data showed a significant BMI-related increase in risk of receptor-negative breast cancers. As expected, the inverse relationship of excess body weight with risk of ER+PR+ breast cancer among women younger than 49 years progressively turned into a direct relationship among women of more advanced age. Furthermore, we observed increases in the risks of both hormone receptor-negative and -positive tumors among women who used HRT at baseline, although the increase in risk was weaker for ER-PR- than for ER+PR+ tumors. The HRT-related increases in risks of both ER-PR- and ER+PR+ breast cancer were stronger among the leaner women, and this increase was independent of HRT type. Finally, with respect to measures of body fat distribution (waist and hip circumferences) no relationships with either receptor-negative or -positive breast cancer were observed after adjustment for BMI.

Cancer registry data show that overall breast cancer incidence increases with age. However, after the age of 50, rates of hormone receptor-negative disease no longer increase with advancing age, whereas the incidence rates of ER-positive tumors continue, albeit at a reduced pace compared to earlier ages [29-33]. This age-related pattern of incidence rates of ER-PR- and ER+PR+ tumors was also apparent in our data. The change in the age-related rise in incidence rates after age 50 suggests a possible relationship of both receptor-negative and -positive breast cancer with the menopause-related cessation of ovarian estrogen and/or progesterone synthesis [29]. While a wide array of epidemiologic, clinical and experimental evidence has clearly established a late-stage growth-promoting effect of estrogens, especially on estrogen-sensitive tumors [34], there is substantial evidence that estrogens may also play an important role in earlier evolutionary stages of the development of both ER-negative and -positive tumor types [29,35,36]. Indeed, evidence suggests that a large proportion of ER-negative tumors may arise from estrogen-responsive precursor tumors or cells and that estrogen sensitivity may be lost at later stages of tumor development [35]. In addition, mammary stem cells have been shown to be responsive to sex steroid hormones despite not having a clear expression of an ER or PR [37].

For ER-PR- tumors, our findings across the age-bands are consistent with those from the meta-analysis by Suzuki *et al*. (2009) [6] of nine cohort and twenty-two case control studies, which also showed no association of BMI with risk of ER-PR- tumors in either premenopausal (1481 cases) or postmenopausal women (1522 cases). However, when our analysis was restricted to postmenopausal women who never used HRT, BMI was associated with ER-PR- tumors. A recent analysis of the Nurses' Health Study II cohort showed a more than two-fold increase in risk of ER-negative premenopausal breast cancer (*n *= 131) for women in higher categories of BMI, waist and hip circumferences [38]. In addition, an analysis within the Women's Health Initiative cohort [39] showed a moderate increase in risk of triple-negative (ER-/PR-/HER2-) breast tumors among postmenopausal women who had more elevated BMI (HR = 1.35 (95%CI = 0.92 to 1.99) for the top versus bottom quartiles of BMI).

For receptor-positive tumors, our findings on the relationships of risk with BMI are well in line with those from previous epidemiologic studies. The meta-analysis by Suzuki *et al*. showed a 33% increase in risk of ER+PR+ tumors per five-unit increase in BMI among postmenopausal women (5,469 cases; risk estimate (RE) = 1.33 (95%CI = 1.20 to 1.48)), and a 10% reduction in risk of ER+PR+ disease among premenopausal women (2,643 cases; RE = 0.90 (95%CI = 0.82 to 0.99)) [6]. Furthermore, similar to our observations, this meta-analysis showed a somewhat stronger association of BMI with risk of hormone ER+PR+ tumors for women who did not use HRT [6]. Finally, still in line with our findings, one recent prospective study also showed an inverse relationship of BMI with breast cancer risk in premenopausal women that was restricted to ER-positive tumors [40], and another recent study showed that the direct risk association of BMI with ER-positive cancers was stronger among postmenopausal women who did not use HRT [39].

The direct risk association of general obesity with both ER-PR- and ER+PR+ breast cancer in postmenopausal women not using HRT, may be explained by adiposity-related increases in circulating estrogen levels, due to conversion of androgens into estrogens within adipose tissue [41]. The restriction of BMI-related risks to HRT nonusers is plausible, because HRT use is a dominant source of circulating estrogens and progestins in postmenopausal women [42] that augments blood concentrations to levels equaling those in premenopausal women, compared to which adiposity-related effects on endogenous synthesis are small.

The mechanisms that underlie the inverse association of BMI with risk of ER+PR+ tumors among women of reproductive age are still largely unclear [43]. Although it has been speculated that obesity-related reductions in circulating estrogen levels and/or reductions in circulating progesterone (due to chronic anovulation) could play a role [44], epidemiological studies of breast cancer risk in relation to premenopausal blood levels of progesterone and estrogens provide no direct support for this [44,45].

An important observation in our study is that current users of HRT, compared to women who did not use HRT, had higher relative risks of both receptor-negative and -positive, but with a stronger increase in risk for the ER+PR+ subtype. Some previous prospective studies have documented similar increases in risks of both ER-negative and -positive breast tumors [46,47], especially among users of combined estrogen-plus-progestin regimens [47-49], whereas other studies showed such increases only for ER-positive disease [9,50].

Another significant observation in our study was that for both breast cancer subtypes the increased risk association in current HRT users depended upon a women's BMI. For receptor-negative and -positive breast cancers, HRT-related increases in risk were stronger among the leaner women and was not restricted to a particular HRT type. Switching perspectives, this statistical interaction also manifested itself in a lack of BMI-related breast cancer risks for both receptor-negative and -positive tumors among current HRT users. A previous analysis of the EPIC data [14] and several other studies [6,10-13,50,51] have shown an inverse association of BMI with the risk of breast cancer overall among HRT users and, from our present analysis, it appears that this overall inverse relationship could be attributable to a relative attenuation of the HRT-related increase in risk in receptor-negative and -positive tumors among more obese women. The endocrine or other mechanisms that may underlie this attenuation of the HRT-related risk remains elusive.

The stronger association of HRT use in lean women in comparison to more overweight women could be potentially related to the different patterns of HRT use. As expected, we observed, a higher proportion of current use in lean women. Within past users of HRT, women of a higher BMI tended to have a shorter cumulative duration of HRT use. Although data was restricted to information collected at baseline, risk estimates in past and current users were not affected by adjustment for duration of use (data not shown).

It is important to acknowledge the limitations of our study. The determination of ER and PR status in breast tumors is a standard part of breast cancer diagnosis used to predict endocrine therapy response [3]. Nevertheless, while a number of studies have shown that the classification of the ER and PR in tumors is relatively robust [52,53], the accuracy of classifying an ER- or PR-negative tumor remains controversial [54,55]. Within the EPIC cohort, as in most other studies, the classification of breast cancer as receptor-positive and -negative (ER, PR or joint ERPR) is based upon a variety of laboratory assay types and quantification methods, reflecting pathology practices across Europe and time. Recently, recommendations were made to lower the threshold of the percentage of stained cells from ≥10% to ≥1% to indicate receptor-positive tumors [56,57], and some of our breast cancer cases classified as ER-negative or PR-negative might have been actually receptor-positive tumors according to these newest criteria. A further limitation of our study is the lack of prospective follow-up information of both BMI and HRT use after recruitment.

Major strengths of the present study are its prospective design and large number of incident cases with receptor information. This large number of cases allowed in-depth analyses of BMI-related relative risk patterns across age bands, describing risk associations and their progressive changes from premenopausal to late postmenopausal age. Furthermore, the large case numbers allowed the examination of interaction effects between excess body weight and use of postmenopausal HRT and by HRT type.

## Conclusion

In summary, we found for receptor-negative tumors, increased BMI among postmenopausal women was associated with risk of receptor-negative tumors, however, only in never users of HRT. In previous studies, excess body weight was associated with an inverse risk only of receptor-positive breast cancer in women aged less than 49, which progressively turned into an increased risk of receptor-positive disease as women aged. Furthermore, women who reported current HRT use at baseline were at an increased risk of both receptor-negative and -positive breast cancer, although this increase was stronger for receptor-positive tumors. In both receptor-negative and -positive breast cancers our data show a stronger HRT-related increase in risk among leaner than among more obese women. This latter interaction also meant that BMI-related increases in risks of receptor-negative and -positive breast cancers were observed among nonusers of HRT only, whereas a possible BMI-related relative decrease (receptor-negative disease) or no increase in risk (receptor-positive disease) may be seen among current users of HRT.

## Abbreviations

BMI: body mass index; CI: confidence interval; EPIC: European Prospective Investigation into Cancer and Nutrition; ER: estrogen receptor; HR: hazard ratio; HRT: hormone replacement therapy; OC: oral contraceptives; PR: progesterone receptor; RE: risk estimate.

## Competing interests

The authors declare that they have no competing interests.

## Authors' contributions

RR, AL, LD and RK contributed to the conception of the current analysis and all authors were involved in the design and acquisition of data from the EPIC cohort. RR, AL, LD and RK contributed to the analysis and all authors contributed to the interpretation of the data. RR, AL and RK drafted the manuscript and all authors revised the final draft critically for important critical content. All authors have given final approval of the version to be published.

## Supplementary Material

Additional file 1 **Proportions and patterns of postmenopausal HRT use across BMI tertiles^1^**. ^1^BMI tertile 1: < 22.5 kg/m^2^; BMI tertile 2: 22.6 to 25.8 kg/m^2^; BMI tertile 3: > 25.9 kg/m^2^. Duration and time since HRT use are for past users of HRT, as reported at baseline recruitment. Click here for file

Additional file 2 **Hazard ratios of ER-positive and ER-negative tumors for increased BMI across five-year age bands**. All models are for a 5 kg/m^2 ^increase in BMI and were stratified by age at recruitment and study center. Hazard ratio estimates are shown for all women and HRT never users. Click here for file

Additional file 3 **Hazard ratios of PR-positive and PR-negative tumors for increased BMI across five-year age bands**. All models are for a 5 kg/m^2 ^increase in BMI and were stratified by age at recruitment and study center. Hazard ratio estimates are shown for all women and HRT never users. Click here for file

Additional file 4 **Hazard ratios of joint ER+PR+ and ER-PR- tumors for increased waist circumference across five-year age bands**. All models are for a 5 kg/m^2 ^increase in BMI and were stratified by age at recruitment and study center. Hazard ratio estimates are shown for all women and HRT never users. Click here for file

Additional file 5 **Hazard ratios of joint ER+PR+ and ER-PR- tumors for increased hip circumference across each age band**. All models are for a 5 kg/m^2 ^increase in BMI and were stratified by age at recruitment and study center. Hazard ratio estimates are shown for all women and HRT never users. Click here for file

Additional file 6 **Hazard ratios of ER+PR+ and ER-PR- tumors for postmenopausal estrogen plus progestin HRT user categories within tertiles of BMI^2^**. Hazard ratios of ER+PR+ and ER-PR- tumors for estrogen plus progestin HRT user categories within tertiles of BMI. All models are stratified by age at recruitment and EPIC center. BMI tertile 1: < 22.5 kg/m^2^; BMI tertile 2: 22.6 to 25.8 kg/m^2^; BMI tertile 3: > 25.9 kg/m^2^. Estrogen plus progestin HRT use at baseline recruitment. Assessment for interaction between BMI and HRT user categories was calculated using the log likelihood ratio test for models with and without the interaction term for HRT never and current use by BMI tertiles. Click here for file

Additional file 7 **Hazard ratios of ER+PR+ and ER-PR- tumors across levels of BMI within categories of postmenopausal estrogen plus progestin HRT users**. Hazard ratios of ER+PR+ and ER-PR- tumors across BMI tertiles and per 5 kg/m^2 ^in estrogen plus progestin HRT user categories. All models are stratified by age at recruitment and EPIC center. BMI tertile 1: < 22.5 kg/m^2^; BMI tertile 2: 22.6 to 25.8 kg/m^2^; BMI tertile 3: > 25.9 kg/m^2^. Click here for file

Additional file 8 **Hazard ratios of ER+PR+ and ER-PR- tumors for postmenopausal estrogen only HRT user categories within tertiles of BMI**. Hazard ratios of ER+PR+ and ER-PR- tumors for estrogen-only HRT user categories within tertiles of BMI. All models are stratified by age at recruitment and EPIC center. BMI tertile 1: < 22.5 kg/m^2^; BMI tertile 2: 22.6 to 25.8 kg/m^2^; BMI tertile 3: > 25.9 kg/m^2^. Estrogen-only HRT use at baseline recruitment. Assessment for interaction between BMI and HRT user categories was calculated using the log likelihood ratio test for models with and without the interaction term for HRT never and current use by BMI tertiles. Click here for file

Additional file 9 **Hazard ratios of ER+PR+ and ER-PR- tumors across levels of BMI within categories of postmenopausal estrogen only HRT users**. Hazard ratios of ER+PR+ and ER-PR- tumors across levels of BMI tertiles and per 5 kg/m^2 ^in estrogen-only HRT user categories. All models are stratified by age at recruitment and EPIC center BMI tertile 1: < 22.5 kg/m^2^; BMI tertile 2: 22.6 to 25.8 kg/m^2^; BMI tertile 3: > 25.9 kg/m^2^. Click here for file

Additional file 10 **Hazard ratios of ER+PR+ and ER-PR- tumors across BMI tertiles within E+P^1 ^HRT user categories**. ^1^Combined estrogen and progesterone HRT. All models were restricted to postmenopausal women with information on baseline HRT use and stratified by age at recruitment and study center. HRT never users within BMI tertile1 were used as the reference category. BMI tertile 1: ≤22.5 kg/m^2^; BMI tertile 2: 22.6 to 25.8 kg/m^2^; BMI tertile 3: ≥25.9 kg/m^2^. Click here for file

Additional file 11 **Hazard ratios of ER+PR+ and ER-PR- tumors across BMI tertiles within E-only^1 ^HRT user categories**. ^1^Estrogen-only HRT. All models were restricted to postmenopausal women with information on baseline HRT use and stratified by age at recruitment and study center. HRT never users within BMI tertile1 were used as the reference category. BMI tertile 1: ≤22.5 kg/m^2^; BMI tertile 2: 22.6 to 25.8 kg/m^2^; BMI tertile 3: ≥25.9 kg/m^2^. Click here for file
