# Supplementary material for: Adiposity, hormone replacement therapy use and breast cancer risk by age and hormone receptor status: a large prospective cohort study
Source: Breast Cancer Res. 2012 May 14;14(3):R76. doi: 10.1186/bcr3186 (PMC3446339; doi:10.1186/bcr3186)
Supplement: Additional file 4 — Hazard ratios of joint ER+PR+ and ER-PR- tumors for increased waist circumference across five-year age bands. All models are for a 5 kg/m2 increase in BMI and were stratified by age at recruitment and study center. Hazard ratio estimates are shown for all women and HRT never users. [file bcr3186-S4.PPT]

## Slide 1
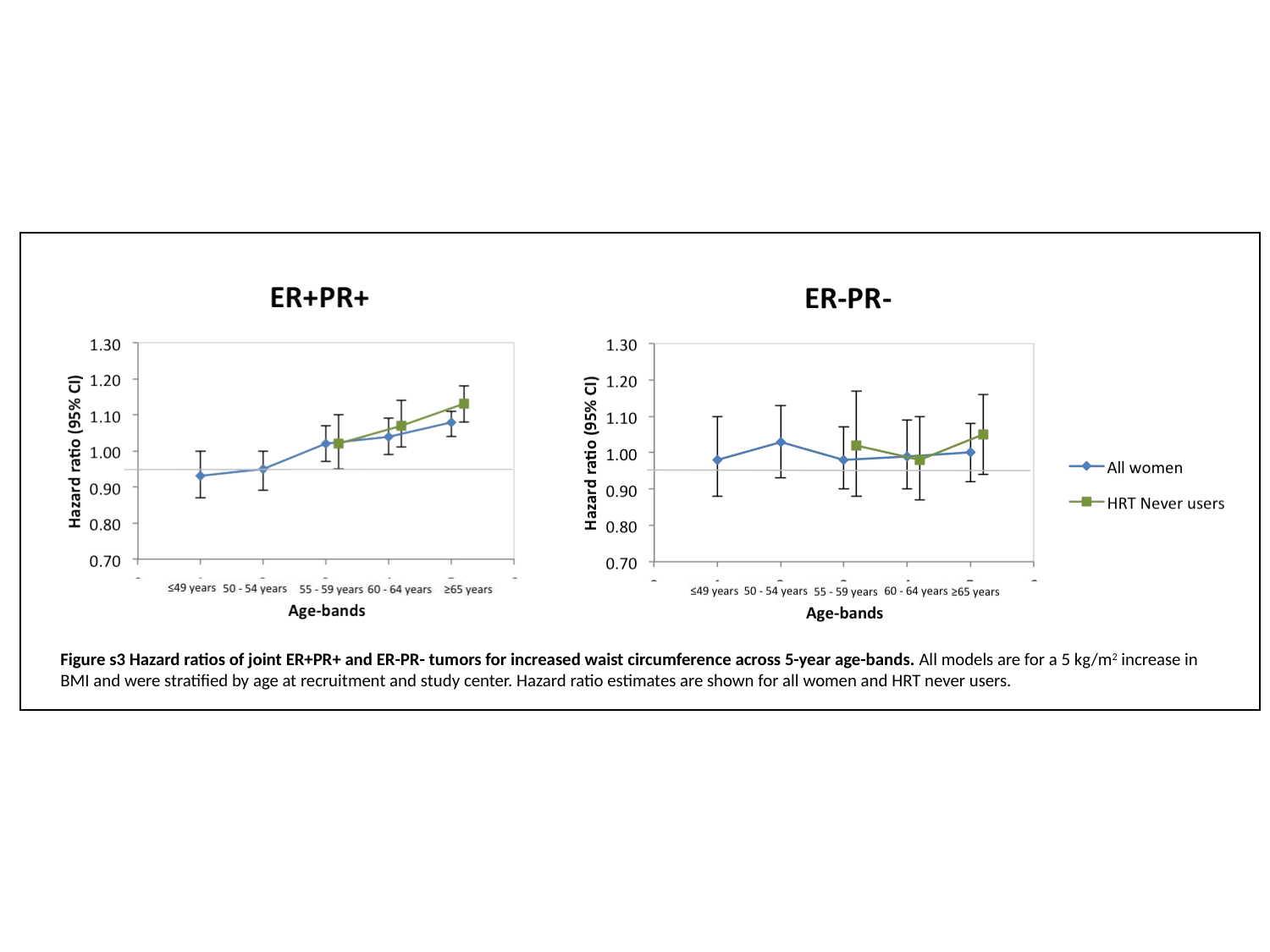

Figure s3 Hazard ratios of joint ER+PR+ and ER-PR- tumors for increased waist circumference across 5-year age-bands. All models are for a 5 kg/m2 increase in BMI and were stratified by age at recruitment and study center. Hazard ratio estimates are shown for all women and HRT never users.
