# Supplementary material for: Adiposity, hormone replacement therapy use and breast cancer risk by age and hormone receptor status: a large prospective cohort study
Source: Breast Cancer Res. 2012 May 14;14(3):R76. doi: 10.1186/bcr3186 (PMC3446339; doi:10.1186/bcr3186)
Supplement: Additional file 10 — Hazard ratios of ER+PR+ and ER-PR- tumors across BMI tertiles within E+P1 HRT user categories. 1Combined estrogen and progesterone HRT. All models were restricted to postmenopausal women with information on baseline HRT use and stratified by age at recruitment and study center. HRT never users within BMI tertile1 were used as the reference category. BMI tertile 1: ≤22.5 kg/m2; BMI tertile 2: 22.6 to 25.8 kg/m2; BMI tertile 3: ≥25.9 kg/m2. [file bcr3186-S10.PPT]

## Slide 1
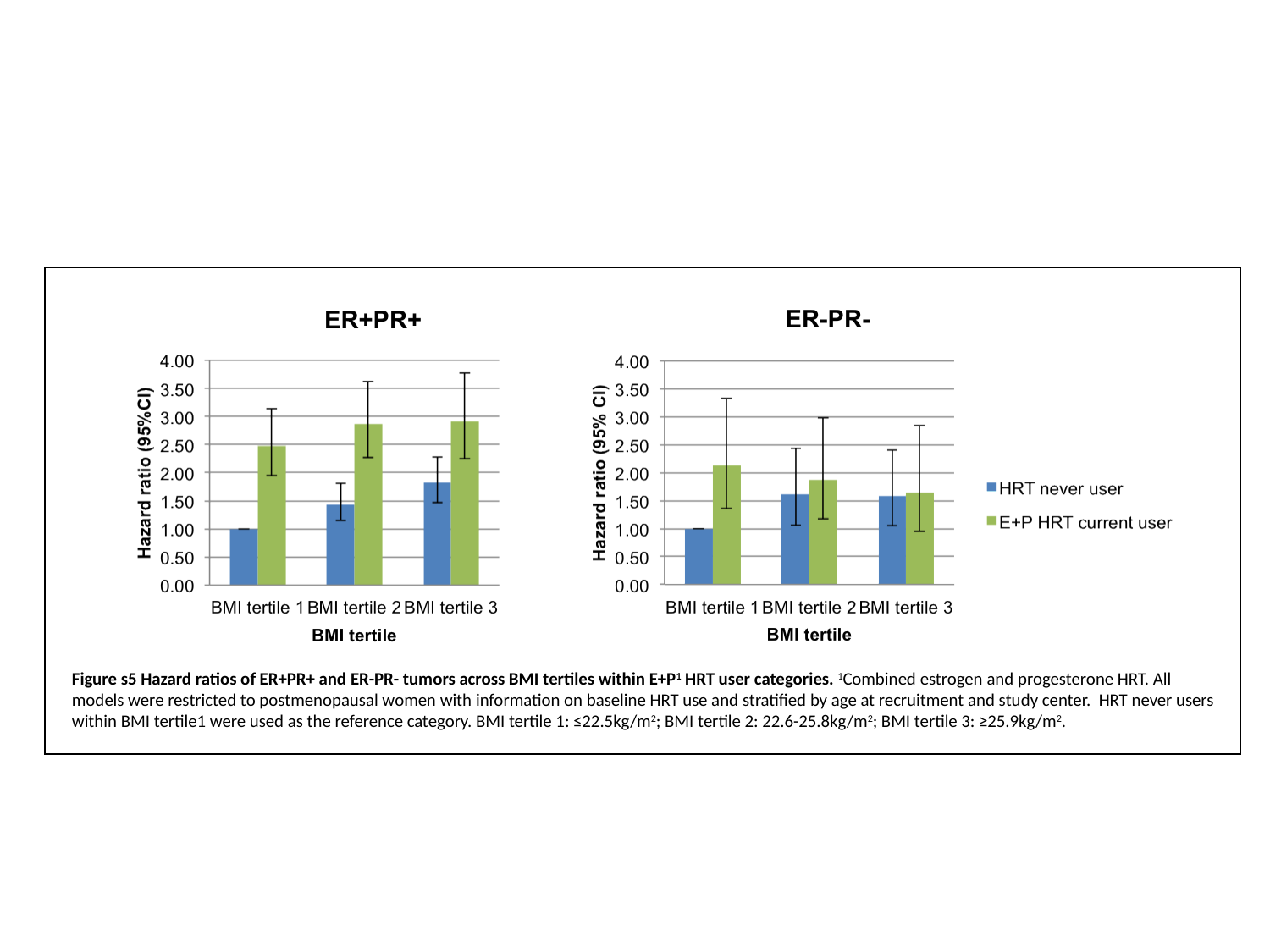

Figure s5 Hazard ratios of ER+PR+ and ER-PR- tumors across BMI tertiles within E+P1 HRT user categories. 1Combined estrogen and progesterone HRT. All models were restricted to postmenopausal women with information on baseline HRT use and stratified by age at recruitment and study center. HRT never users within BMI tertile1 were used as the reference category. BMI tertile 1: ≤22.5kg/m2; BMI tertile 2: 22.6-25.8kg/m2; BMI tertile 3: ≥25.9kg/m2.
