# Supplementary material for: Adiposity, hormone replacement therapy use and breast cancer risk by age and hormone receptor status: a large prospective cohort study
Source: Breast Cancer Res. 2012 May 14;14(3):R76. doi: 10.1186/bcr3186 (PMC3446339; doi:10.1186/bcr3186)
Supplement: Additional file 11 — Hazard ratios of ER+PR+ and ER-PR- tumors across BMI tertiles within E-only1 HRT user categories. 1Estrogen-only HRT. All models were restricted to postmenopausal women with information on baseline HRT use and stratified by age at recruitment and study center. HRT never users within BMI tertile1 were used as the reference category. BMI tertile 1: ≤22.5 kg/m2; BMI tertile 2: 22.6 to 25.8 kg/m2; BMI tertile 3: ≥25.9 kg/m2. [file bcr3186-S11.PPT]

## Slide 1
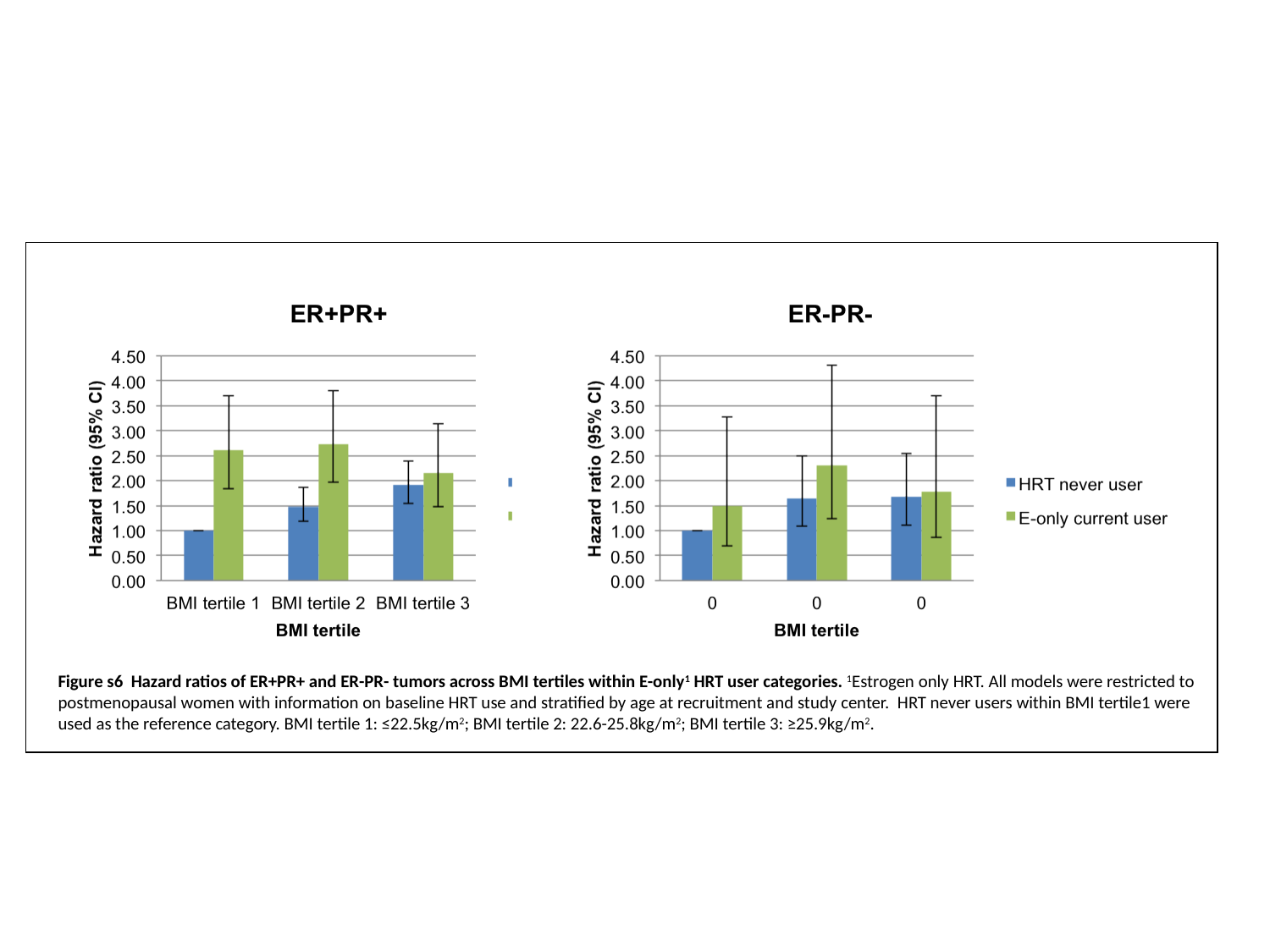

Figure s6 Hazard ratios of ER+PR+ and ER-PR- tumors across BMI tertiles within E-only1 HRT user categories. 1Estrogen only HRT. All models were restricted to postmenopausal women with information on baseline HRT use and stratified by age at recruitment and study center. HRT never users within BMI tertile1 were used as the reference category. BMI tertile 1: ≤22.5kg/m2; BMI tertile 2: 22.6-25.8kg/m2; BMI tertile 3: ≥25.9kg/m2.
